# Prognostic value of hyperuricemia for patients with sepsis in the intensive care unit

**DOI:** 10.1038/s41598-022-04862-3

**Published:** 2022-01-20

**Authors:** Shizhen Liu, Zhihua Zhong, Fanna Liu

**Affiliations:** 1 Department of Nephrology, The First Affiliated Hospital of Jinan University, Guangzhou, 510630 Guangdong China; 2 Department of Nephrology, Jiangmen People’s Hospital, Jiangmen, 529000 Guangdong China; 3 College of Information Science and Technology, Jinan University, Guangzhou, China

**Keywords:** Biomarkers, Diseases, Health care, Medical research

## Abstract

This study evaluated the relationship between hyperuricemia at admission and the clinical prognosis of patients with sepsis. The data were obtained from the Intensive Care Medical Information Database III. The patients were divided into a normal serum uric acid group and a hyperuricemia group. The main outcome was 90-day mortality, and the secondary outcomes were hospital mortality, 30-day mortality, and acute kidney injury. Propensity score matching was used to balance the baseline characteristics of the groups. Our study retrospectively included 954 patients. Before and after propensity score matching, the incidence of AKI, the 30-day and 90-day mortality rates were significantly higher in the hyperuricemia group. Cox regression analysis showed that hyperuricemia was significantly associated with 90-day mortality (HR 1.648, 95% CI 1.215–2.234, *p* = 0.006), and hyperuricemia was significantly associated with the incidence of AKI (HR 1.773, 95% CI 1.107–2.841, *p* = 0.017). The Kaplan–Meier survival curve showed that the 90-day survival rate was significantly lower in the hyperuricemia group. In patients with sepsis in the intensive care unit, hyperuricemia was significantly associated with increased risk 90-day all-cause mortality and the incidence of AKI.

## Introduction

Hyperuricemia is defined as a serum uric acid level > 6.0 mg/dl in females or > 7.0 mg/dl in males^1^. According to the 2016 National Health and Nutrition Examination Survey (NHANES) data, the prevalence of hyperuricemia in the United States has reached 20.1%^2^. Uric acid is the final product of purine catabolism by xanthine oxidase in liver cells, accounting for more than 50% of the total antioxidant activity in the blood, and it delays cell senescence through its antioxidant effect^3–5^. A study has also showed that serum uric acid delays the progression of cardiovascular disease through its antioxidant activity^4^. However, serum uric acid stimulates not only the production of NADPH oxidase, growth factor, cyclooxygenase 2 and thromboxane but also plasma renin activity and renin expression, resulting in oxidative stress^6–9^. Hyperuricemia will promote endothelial cell senescence and apoptosis by increasing expression of the renin-angiotensin system (RAS)^10^. At the same time, oxidative stress increases the generation and elimination of reactive oxygen species (ROS) in cells, and causes damage to vascular endothelial cells^11^. ROS accumulates in cells, triggers endoplasmic reticulum stress, inhibits the activity of endothelial nitric oxide synthase (eNOS) through a PKC-dependent pathway, and the reduction of NO further aggravates endothelial dysfunction^12^. In addition, hyperuricemia will form sodium urate crystals in the vascular intima, increase the expression of leukocyte adhesion molecules and stimulate the production of a large number of related inflammatory factors, causing endothelial cell dysfunction. These changes promote the occurrence of inflammatory reactions, amplify the inflammatory process, and enhance immunity cell interactions eventually induce and aggravate the progression of sepsis^13,14^. Meanwhile, we found that hyperuricemia is associated with the prognosis of many diseases. A meta-analysis showed that an elevated blood uric acid level is an independent risk factor for heart failure and adverse consequences in patients with pre-existing heart failure^15^. Hyperuricemia can predict one-year mortality in patients with acute heart failure and adverse outcomes in and the mortality of patients with acute myocardial infarction^16,17^. Hyperuricemia is an independent risk factor for chronic kidney disease (CKD)^18^. A study reported that elevated levels of uric acid in patients with sepsis are associated with an increased risk of acute kidney injury (AKI) and acute respiratory distress syndrome (ARDS)^19^. However, a study showed that the serum uric acid level on admission to the intensive care unit (ICU) was not associated with the long-term prognosis in critically ill patients^20^. Sepsis causes approximately 30% to 50% of the short-term mortality in the ICU, and it is one of the primary challenges in the ICU^21^. At present, there have been few articles on the association between the hyperuricemia and long-term prognosis of sepsis patients in the ICU. The studies that have been performed have had small sample sizes and inconsistent conclusions. The main purpose of this study was to explore the prognostic value of hyperuricemia in ICU patients with sepsis.

## Materials and methods

### Data source

The Medical Information Mart for Intensive Care III (MIMIC-III) (https://physionet.org/content/mimiciii/1.4/) is a public free ICU database that contains data from more than 50,000 ICU patients who visited the Beth Israel Deaconess Medical Center in the United States from 2001 to 2012^22^. The author (Zhihua Zhong) obtained access to the database and extracted the relevant clinical data, including patient demographic data and laboratory results. The use of the database was approved by the Institutional Review Boards of Beth Israel Deaconess Medical Center (Boston, MA) and the Massachusetts Institute of Technology (Cambridge, MA, USA). The patient's information has been standardized and the project did not affect clinical care, so requirement for individual patient consent was waived. The project was conducted in accordance with the Declaration of Helsinki (as revised in 2013).

### Inclusion and exclusion criteria

The database has data from a total of 58,976 patients admitted to the ICU^22^. Adult patients who met the diagnostic criteria for sepsis and AKI were eligible for inclusion in the study. The diagnosis of sepsis was based on the Sepsis-3 guidelines^23^, namely, a sequential organ failure assessment (SOFA) score ≥ 2 within 24 h of admission with at least one site of infection. The diagnosis of AKI was based on the kidney disease: improving global outcomes (KDIGO) guidelines^24^, and AKI was diagnosed after enter in ICU. The following were the exclusion criteria: (1) age less than 18 years; (2) no serum uric acid data within 24 h after admission to the ICU; (3) ICD code is chronic kidney disease; (4) ICD code is gout. Ultimately, 954 patients were included in this study. The patients included in the study were divided into a hyperuricemia group and a normal serum uric acid level group according to their serum uric acid levels on the first day of admission.

### Data extraction

We extracted the data with structured query language (SQL) in PostgreSQL. We extracted the following data: demographic information, vital signs, comorbidities, laboratory parameters, and scores. The demographic information extracted were age, sex and race. The vital signs extracted were body mass index (BMI), mean arterial pressure (MAP), respiratory rate, heart rate and body temperature. The comorbidities investigated in this study were coronary artery disease (CAD), CKD, chronic obstructive pulmonary disease (COPD), diabetes, hypertension, and liver disease (including ICD-9 code: 5719, 5728, 5718). The following laboratory parameters were extracted: the white blood cell (WBC) count; the platelet count; the levels of blood potassium, blood sodium, blood creatinine, blood urea nitrogen (BUN), serum albumin, blood sugar, lactate level, serum uric acid; the pH, the partial pressure of oxygen (pO_2_), the partial pressure of carbon dioxide (pCO_2_), bilirubin and prothrombin time(PT). The SOFA score and simplified acute physiology score (SAPS II) score were also extracted. Hyperuricemia is defined as a serum uric acid level > 6.0 mg/dl in females or > 7.0 mg/dl in males.

### Outcomes

The main outcome of this study was 90-day mortality, while the secondary outcomes were AKI, hospital mortality and 30-day mortality.

## Statistical analysis

Continuous variables were expressed as the means ± standard deviations or interquartile ranges (IQRs) that were normal distribution and not subject to normal distributed variables, respectively. Classification variables are expressed as totals and percentages. The chi-square test was used to compare classified variables between groups. Wilcoxon rank-sum test and Student t test were used for continuous variables that were not subject to normal distribution and normal distributed variables, respectively.

To reduce the imbalance in baseline characteristics between the hyperuricemia group and the normal serum uric acid level group, we used a propensity score matching ratio of 1:1 with a caliper value of 0.03. The selection of variable for PSM included ethnicity, gender, first care unit, length of stay, age, sofa, use of vasopressin, saps II, heart rate, respiratory rate, temperature, hemoglobin, lactate, pco2, PH, po2, potassium, COPD, coronary heart disease, diabetes, hypertension, liver disease, creatinine, glucose, platelet, potassium, sodium, urea nitrogen, white blood cell, albumin, mean arterial pressure, BMI. The outcomes were compared before and after matching. Multivariate Cox regression was used to analyze the risk factors for 90-day mortality in patients with septicemia in the ICU, while logistic regression was used to analyze the risk factors for the incidence of AKI. When variables had a *p* value < 0.05 in the univariate analyses were included in the multivariable analysis. The model of Cox multivariate regression included hyperuricemia group, age, saps II, heart rate, temperature, pco2, po2, potassium, platelet, bilirubin, and prothrombin time (PT). Kaplan–Meier survival analysis was used to determine the difference in 90-day mortality between the hyperuricemia group and the normal serum uric acid level group. We conducted a stratified analysis to determine whether serum uric acid levels were associated with 90-day mortality in subgroups stratified by age, gender, AKI, diabetes, hypertension, and SOFA scores. At the same time, it was also determined whether serum uric acid levels were associated with AKI occurrence in subgroups stratified by age, gender, hypertension, SOFA scores, bilirubin and PT. *P* < 0.05 was considered statistically significant. All statistical analyses were carried out using Stata software.

## Results

### Patient characteristics

**Figure 1:**
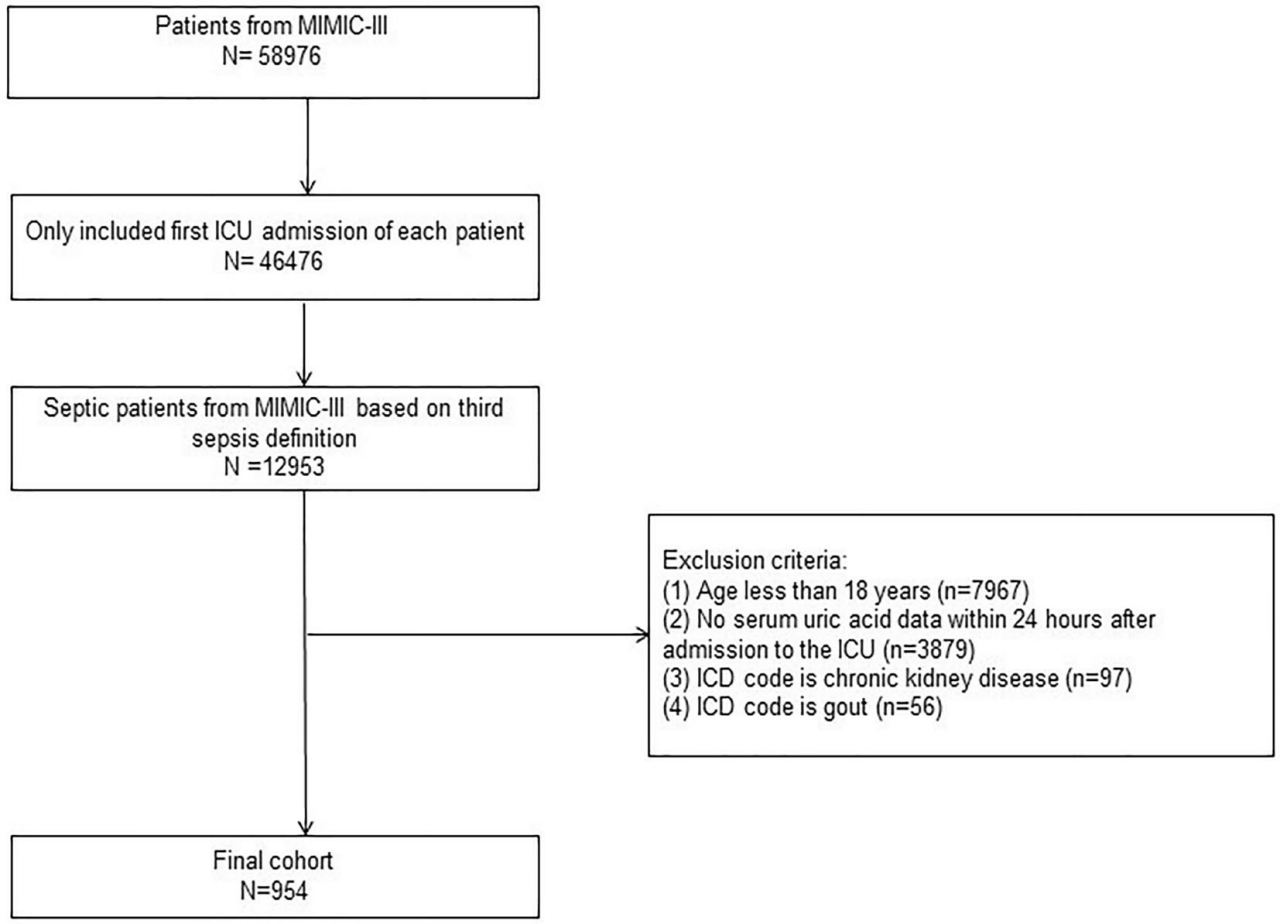
Flow diagram of the study. *ICU* Intensive Care Unit; *ICD* International Classification of Diseases.

A total of 954 patients were enrolled in this study and were divided into the hyperuricemia group (n = 345) and the normal serum uric acid level group (n = 609) (Fig. 1). Before propensity score matching, there were significant differences in age, race, body temperature, MAP, BMI, COPD, diabetes, SOFA score, SAPS II score, WBC counts, PLT counts, serum potassium levels, serum creatinine levels, BUN levels, pH, PT and uric acid between the two groups (Table 1). PSM (1:1 matching) was performed, and 225 patients in the normal serum uric acid level group were matched with 225 patients in the hyperuricemia group. After matching, the degree of imbalance between the two groups decreased significantly, and the baseline characteristics between the two groups were comparable.

**Table 1:** Baseline characteristics before and after propensity-score matching.

Characteristics	Before matching				After matching			
	Normal serum uric acid group	Hyperuricemia group	SMD	*p* value	Normal serum uric acid group	Hyperuricemia group	SMD	*p* value
Clinical parameters, n	609	345			225	225		
Age (years)	63.1 (49.1, 74.7)	65.7 (54.0, 76.9)	0.2	0.003	68.2 (55.0, 77.5)	65.0 (54.2, 77.5)	< 0.1	0.337
Male, n(%)	356 (37.3)	184 (19.2)		0.142	110 (24.4)	120 (26.7)		0.396
**Ethnicity, n (%)**								
White	99 (10.4)	82 (8.6)		0.006	51 (11.3)	55(11.6)		1.0
Black	27 (2.8)	3 (0.3)		0.005	5 (1.1)	3 (0.7)		0.721
Yellow	436 (45.7)	233 (24.4)		0.214	157 (34.9)	155 (34.4)		0.916
Temperature (°C)	37.0 (36.5, 37.5)	36.8 (36.6, 37.3)	0.3	< 0.001	36.8 (36.4, 37.3)	36.9 (36.4, 37.4)	< 0.1	0.451
Heart rate (bpm)	92.1 (80.3, 105.9)	90.6 (81.0, 102.6)	< 0.1	0.229	91.8 (79.8, 104.8)	89.8 (79.8, 103.5)	< 0.1	0.312
Respiratory rate (bpm)	19.4 (16.5, 23.0)	19.9 (16.8, 23.0)	< 0.1	0.166	19.9 (16.5, 23.0)	19.9 (16.7, 22.9)	< 0.1	0.343
MAP (mmHg)	77.6 (72.0, 85.9)	75.9 (69.7, 84.5)	< 0.1	0.015	75.8 (70.2, 84.8)	76.5 (70.8, 84.5)	< 0.1	0.291
BMI (kg/m^2^)	30.5 (26.4, 30.5)	30.5 (28.4, 30.7)	0.1	< 0.001	30.5 (27.3, 30.5)	30.5 (27.6, 30.5)	< 0.1	0.095
**Comorbidities, n (%)**								
COPD	5 (0.5)	12 (1.2)		0.006	5 (1.1)	4 (0.9)		1.0
Coronary artery disease	72 (7.5)	55 (5.8)		0.089	40 (8.9)	34 (7.6)		0.525
Diabetes	112 (11.7)	102 (10.7)		< 0.001	58 (12.9)	56 (12.4)		0.914
Hypertension	218 (22.9)	112 (11.7)		0.333	89 (19.8)	76 (16.9)		0.240
Liver disease	8 (0.8)	6 (0.6)		0.807	3 (0.7)	3 (0.7)		1.0
SOFA score	5 (4, 8)	7.0 (5.0, 9.0)	0.3	< 0.001	6 (4, 9)	6 (4, 9)	< 0.1	0.296
SAPS II score	41 (33, 51)	44.0 (37.0, 56.0)	0.3	< 0.001	43 (33, 54)	42 (36, 55)	< 0.1	0.311
**Laboratory tests**								
WBC (× 10^9^/L)	10.8 (5.9, 17.4)	13.1 (8.3, 18.5)	0.1	< 0.001	12.4 (6.7,19.6)	12.5 (8.0, 18.2)	< 0.1	0.468
Platelet(× 10^9^/L)	152 (83, 253)	175 (95, 267)	0.1	0.020	174 (92, 261)	174 (89, 252)	< 0.1	0.324
Potassium (mmol/L)	3.9 (3.6, 4.4)	4.3 (3.7, 4.6)	0.3	< 0.001	4.3 (4.1, 4.4)	4.3 (4.1, 4.4)	< 0.1	0.438
Sodium (mmol/L)	138 (135, 141)	138 (134, 141)	< 0.1	0.491	138 (135, 142)	138 (134, 141)	< 0.1	0.163
Creatinine (mg/dL)	0.9 (0.7, 1.4)	1.8 (1.2, 3.2)	0.7	< 0.001	1.1 (0.8, 2.0)	1.5 (1.0, 2.1)	< 0.1	0.002
Bun (mg/dL)	19 (13, 30)	40 (25, 63)	0.8	< 0.001	27 (18, 45)	32 (22, 47)	< 0.1	0.045
Albumin (mg/dL)	2.8 (2.7, 2.9)	2.8 (2.6, 3)	< 0.1	0.344	2.8 (2.7, 3.0)	2.8 (2.6, 3.0)	< 0.1	0.311
Glucose(mg/dL)	132 (110, 173)	133 (107, 176)	< 0.1	0.414	136 (113, 176)	135 (110, 170)	< 0.1	0.244
PH	7.4 ± 0.1	7.3 ± 0.1	0.1	0.001	7.4 ± 0.1	7.4 ± 0.1	0.1	0.274
pO_2_ (mmHg)	132.5 (96.2, 157.7)	128.3 (91.0, 155.1)	< 0.1	0.126	120.1 (93.4, 149.3)	130.7 (88.5, 154)	< 0.1	0.284
pCO_2_ (mmHg)	40.0 (35.3, 42.5)	40.0 (34.1, 42.6)	< 0.1	0.194	39.7 (35, 42.5)	39.5 (34.4, 42.3)	< 0.1	0.217
Bilirubin (mg/dL)	0.9 (0.5, 2.3)	1.1 (0.5, 2.3)	< 0.1	0.053	1.0 (0.5, 2.3)	1.1 (0.5, 2.5)	< 0.1	0.190
PT (s)	14.1 (13.3, 15.5)	14.9 (13.7, 17.0)	0.3	< 0.001	14.4 (13.3, 16.2)	14.7 (13.7, 16.4)	< 0.1	0.089
Lactate level (mmol/L)	2.7 (1.7, 2.8)	2.8 (1.7, 3.5)	0.1	0.063	2.8 (1.8, 3.1)	2.8 (1.8, 3.6)	< 0.1	0.261
Uric acid (mg/dL)	3.8 (2.7, 5)	9.0 (7.5, 11.1)	2.8	< 0.001	4.1 (3.0, 5.3)	8.6 (7.3, 10.6)	2.6	< 0.001

MAP mean arterial pressure, BMI body mass index, COPD Chronic obstructive pulmonary disease, WBC white blood cell, Bun blood urea nitrogen, pO2 partial pressure of oxygen, pCO2 partial pressure of carbon dioxide, PT prothrombin time. Note: Chi-square test and Wilcoxon rank-sum test were used to compare the differences of categorical and continuous variables respectively.

### Outcome comparisons

Before matching, in-hospital mortality rate, 30-day mortality rate and 90-day mortality rate in the hyperuricemia group were significantly higher than those in the normal uric acid level group. After matching, the incidence of AKI, the 30-day and 90-day mortality rate in the hyperuricemia group were significantly higher than those in the normal serum uric acid level group (Table 2).

**Table 2:** Clinical outcomes before and after propensity-score matching population.

Clinical outcomes	Before matching			After matching		
	Normal serum uric acid group	Hyperuricemia group	*p* value	Normal serum uric acid group	Hyperuricemia group	*p* value
AKI, (n, %)	198 (20.8)	113 (11.8)	0.996	66 (14.7)	86 (19.1)	0.046
Hospital mortality (n, %)	129 (13.5)	114 (11.9)	< 0.001	62 (13.8)	76 (16.9)	0.184
30-day mortality (n, %)	169 (17.7)	139 (14.6)	< 0.001	72 (16)	97 (21.6)	0.019
90-day mortality (n, %)	207 (21.7)	158 (16.6)	< 0.001	82 (18.2)	107 (23.8)	0.022

*AKI* acute kidney injury.

Chi-square test and Wilcoxon rank-sum test were used to compare the differences of categorical and continuous variables respectively.

### Evaluation of risk factors for 90-day mortality and the incidence of AKI

Multivariate Cox regression analysis showed that hyperuricemia was independent risk factors for 90-day mortality. Hyperuricemia was significantly associated with increased risk 90-day mortality (HR 1.648, 95% CI 1.215–2.234, *p* = 0.006) (Table 3; Supplementary Table S1). Multivariate logistic regression analysis found that hyperuricemia was independent risk factors for the incidence of AKI. Hyperuricemia was significantly associated with increased risk of the incidence of AKI (HR 1.773, 95% CI 1.107–2.841, *p* = 0.017) (Table 4; Supplementary Table S1).

**Table 3:** Cox regression analyses to assess risk factors associated with 90-day all-cause mortality in patients with sepsis.

	HR (95% CI)	*p* value
Age	1.017 (1.004–1.029)	0.008
Temperature	0.711 (0.560–0.903)	0.005
Heart rate	1.015 (1.004–1.026)	0.008
SAPS II score	1.017 (1.001–1.033)	0.033
Platelet	0.998 (0.997–1.000)	0.024
Bilirubin	1.042 (1.006–1.079)	0.021
PT	1.066 (1.018–1.116)	0.006
Potassium	1.388 (1.036–1.858)	0.028
pO_2_	0.996 (0.994–0.999)	0.001
pCO_2_	1.025 (1.006–1.045)	0.010
Hyperuricemia	1.648 (1.215–2.234)	0.006

PT prothrombin time, pO2 partial pressure of oxygen, pCO2 partial pressure of carbon dioxide.

**Table 4:** Logistic regression analyses to assess risk factors associated with AKI in patients with sepsis.

	OR (95% CI)	p value
Length of stay	1.060 (1.033–1.087)	< 0.001
Bun	0.968 (0.951–0.985)	< 0.001
Platelet	0.997 (0.995–0.999)	0.007
Bilirubin	1.101 (1.027–1.180)	0.007
PT	1.096 (1.017–1.182)	0.017
Hyperuricemia	1.773 (1.107–2.841)	0.017

Bun blood urea nitrogen, PT prothrombin time.

### Subgroup Analyses

**Figure 2:**
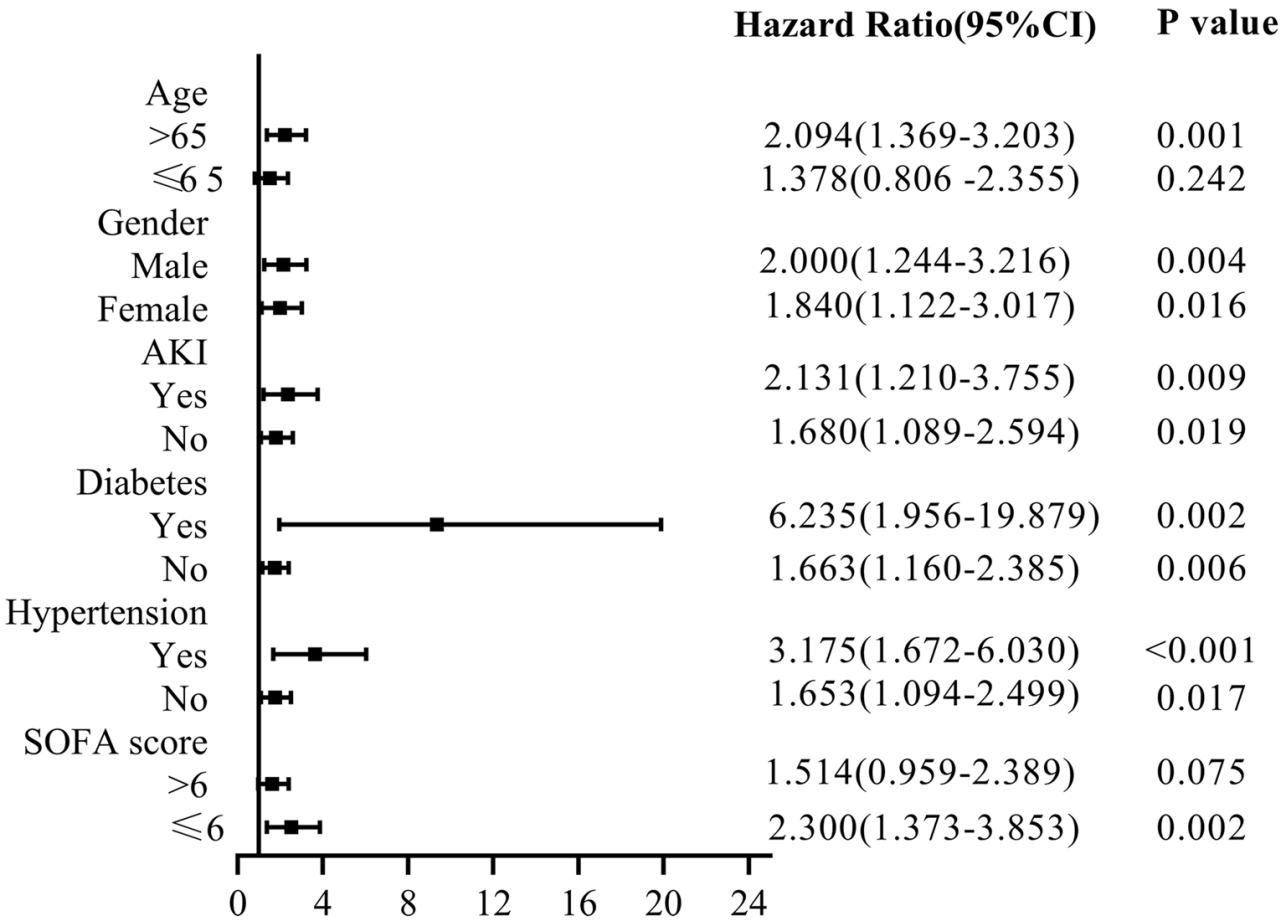
Subgroup analysis of the relationship between hyperuricemia and 90-day all-cause mortality in patients with sepsis.

**Figure 3:**
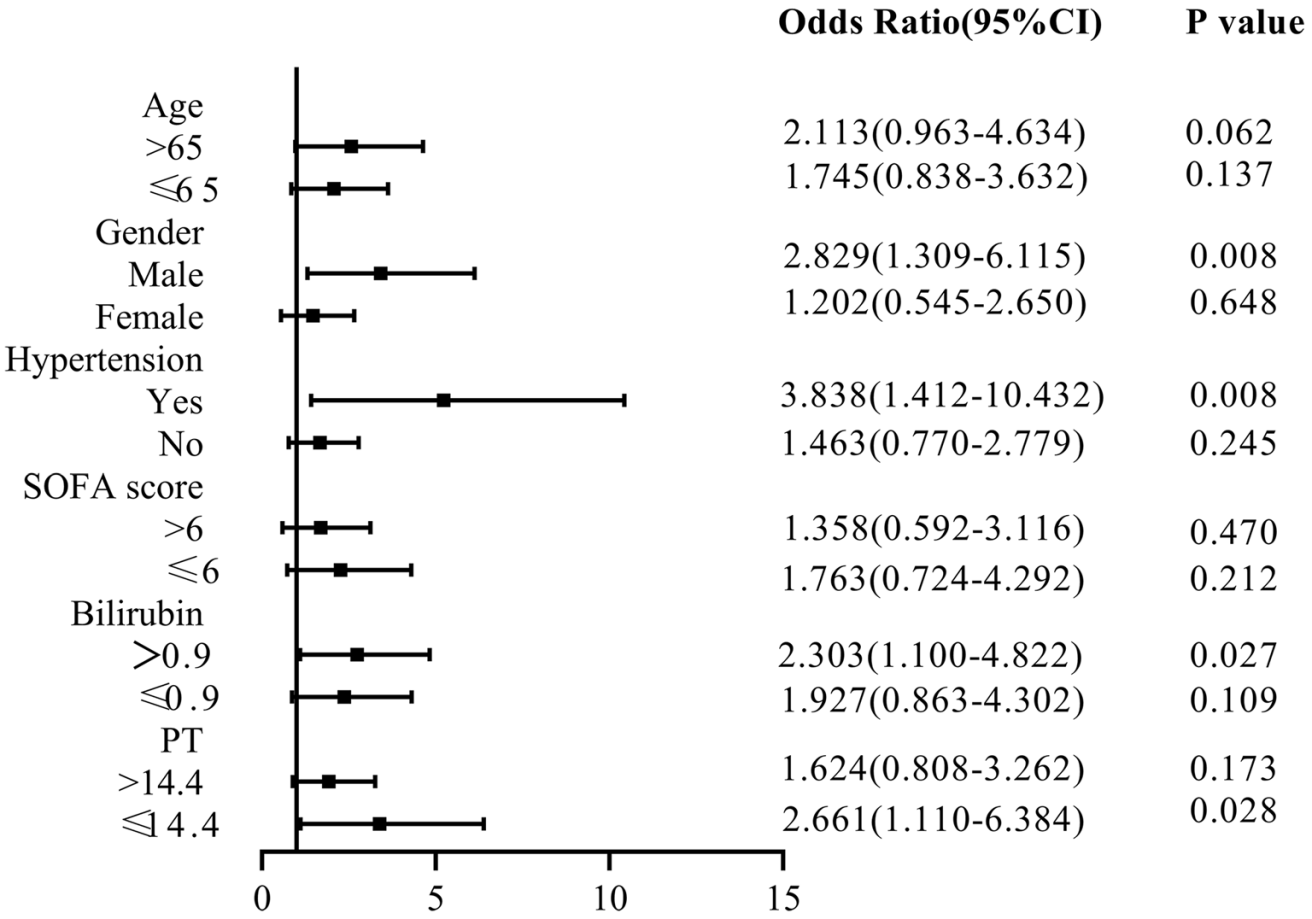
Subgroup analysis of the relationship between hyperuricemia and the incidence of AKI in patients with sepsis.

We performed subgroup analyses to determine the reliability of the relationship between hyperuricemia and the risk of 90-day mortality in patients with septicemia in the ICU (Fig. 2). Significant interactions were observed for age > 65 years, SOFA score ≤ 6 points; Similarly, patients with man (HR 1.695, 95% CI 1.164–2.466), AKI (HR 2.131, 95% CI 1.210–3.755), diabetes (HR 6.235, 95% CI 1.956–19.879) and hypertension (HR 3.175, 95% CI 1.672–6.030) had a significantly higher risk of 90-day hospital mortality with hyperuricemia. The subgroup analysis was performed to assess the reliability of the relationship between hyperuricemia and the risk of AKI in septic patients in the ICU (Fig. 3). Significant interactions were observed for man, bilirubin > 0.9 mg/dL and PT ≤ 14.4 s (*p* < 0.05).

### Kaplan–Meier analysis

**Figure 4:**
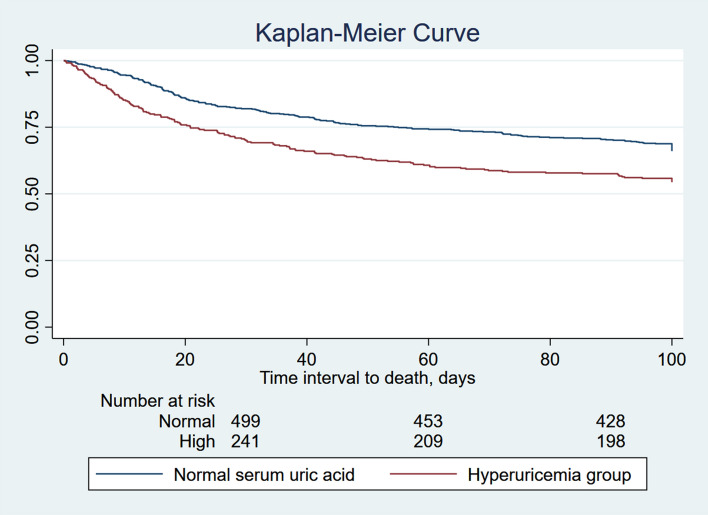
Kaplan–Meier curve was used to evaluate the difference in 90-day all-cause mortality in patients with sepsis with normal serum uric acid levels and hyperuricemia in critical care units. In the Kaplan–Meier analysis, the log-rank test *p* value < 0.001.

The patients were divided into two groups based on their serum uric acid level on admission. The Kaplan–Meier survival curve analysis showed that the 90-day survival rate of patients with normal serum uric acid levels was significantly higher than that of patients with hyperuricemia (log-rank test: *p* < 0.001) (Fig. 4).

## Discussion

Sepsis is one of the main causes of death in the intensive care unit. Its early characteristic is the production of large amounts of reactive oxygen and nitrogen substances, such as superoxide and nitric oxide, which cause tissue damage and lead to DNA decomposition and conversion of purines into uric acid. In patients with severe infections, ischemic and hypoxic organs activate xanthine oxidases that act on xanthine and hypoxanthine, thereby increasing uric acid production. At the same time, severe infections often cause renal damage and reduce uric acid excretion, leading to further uric acid accumulation in the blood^25,26^. Serum uric acid has an antioxidant effect at physiological concentration and has a protective effect on the human body, but hyperuricemia can activate a variety of pro-inflammatory mediators, enhance the activity of endotoxin, and further aggravate the systemic inflammatory response, resulting in tissue damage and even life-threatening effects^27^. As a cell signaling molecule, uric acid affects intracellular signal transduction by promoting the production of ROS and the expression of inflammatory mediators, causing the damage of vascular endothelial cells, thereby aggravating the progression of sepsis^11,28,29^. At present, the possible pathogenic mechanisms of hyperuricemia in sepsis are mainly as follows: (1) RAS system is an important body fluid regulation system in the human body, which maintains the body's metabolic cycle. However, uric acid enters endothelial cells through organic ion transport, activates oxidative stress, up-regulates the expression of angiotensin II, angiotensin receptors 1 and 2, and increases the binding of angiotensin II to the receptors, so that stimulating endothelial cells to produce intercellular cell adhesion molecule-1, IL-1 and other inflammatory factors, which promotes the senescence and apoptosis of endothelial cells^10,30,31^. (2) When uric acid is at a physiological level, it mainly exhibits an antioxidant effect, which can remove some free radicals in the body. However, when uric acid is elevated, it has pro-inflammatory and pro-oxidant effects. Uric acid enters cells through uric acid transporter 1, reducing NADPH oxidase activation, activating the RASS system, and changing mitochondrial energy metabolism to increase oxidative stress, leading to increased intracellular ROS and promoting the expression of inflammatory mediators, thereby destroying proteins, lipids, DNA and RNA to participate in a variety of cellular processes^30,32–34^. (3) The hyperuricemia state can regulate the response of leukocytes to inflammatory modes through epigenetic modifications (including histone methylation), which promotes the release of pro-inflammatory cytokines such as IL-1b and IL-6, and reduce the release of IL-1Ra^13^. When the serum uric acid exceeds the physiological concentration, it is easy to form a kind of sodium urate crystals in the inner cells that behaves as a pro-oxidant molecule, which is sensed by the immune system and promotes the inflammatory process of white blood cells. Uric acid crystals can induce pro-inflammatory cytokines, such as IL-1b, increase the production of ROS, stimulate chemotaxis, and activate NF-kB and mitogen-activated protein kinases pathways^14,35,36^. Uric acid promotes mitochondrial modification of macrophages and increases the production of ROS by mitochondria, and this metabolic change will promote the activation of nucleotide-binding oligomerization domain, leucine-rich repeat and pyrin domain containing-3 (NLRP3) inflammasome and the production of IL-1b, and NLRP3 is a key molecule that connects inflammation and cell metabolism to initiate inflammatory caspase and induce cell-death pathway^36,37^. (4) As the strongest vasodilator in the body, NO plays an important role in the regulation of the cardiovascular system. Hyperuricemia can induce the accumulation of intracellular ROS, trigger endoplasmic reticulum stress, inhibit eNOS activity through PKC-dependent pathways and generate stable 6-aminouracil to reduce NO production, which promote endothelial cell damage^12,38^. Hyperuricemia causes vascular endothelial dysfunction to varying degrees, leading to coagulation dysfunction, inflammatory response, and ischemia–reperfusion injury of tissues and organs, making the infection further aggravated, and internal environmental disorders further increase the level of uric acid, forming Infinite circulation, which causes organ dysfunction and aggravating sepsis.

In a prospective cohort study, there is a significant correlation between serum uric acid and hospital mortality. The serum uric acid level of the dead patients was significantly higher than that of the surviving patients at discharge^39^. In another study, it was found that uric acid can be used as an independent predictor of mortality in hospitalized patients^40^. In a prospective study followed up for 5 years, it was found that elevated uric acid levels in elderly patients were associated with mortality. Compared with patients with uric acid < 7.5 mg/dl, the mortality of patients with uric acid > 7.5 mg/dl was as high as 80%^41^. When the serum uric acid level is higher, the risk of all-cause death and cardiovascular death in elderly patients is significantly higher^42^. The subgroup analysis of this study also found that in patients older than 65 years, hyperuricemia was associated with the 90-day mortality rate. A large-scale cohort study of seven years in Japan found that even a slight increase in serum uric acid levels is an independent risk factor for all-cause and cardiovascular death in men and women^43^. This study also found that hyperuricemia is a risk factor for 90-day mortality in men and women.

It has been reported that hyperuricemia at the time of admission to the cardiac care unit is related to the short-term mortality of patients with myocardial infarction^44^. It has been shown that uric acid can be used as an independent predictor of the prognosis of cardiovascular disease^45^. A study reported significant correlations between elevated serum uric acid levels and hypertension and atherosclerosis. The subgroup analysis in this study also found a significantly elevated risk of 90-day mortality in hyperuricemia patients with hypertension^46^. It has been reported that hyperuricemia is an independent risk factor for mortality in all women with CAD^47^. In this study, subgroup analysis also showed that hyperuricemia increased the risk of 90-day mortality in patients with AKI and diabetes. This is consistent with the following research results. It has been reported that hyperuricemia increases the risks of AKI and all-cause mortality in hospitalized patients^48^. Study had found that there was a significant statistical association between serum uric acid levels and all-cause mortality in people with type 2 diabetes^49^. A study showed that hyperuricemia as an independent predictor of vascular complications and mortality in T2DM patients^50^. A recent study also showed that the level of serum uric acid may be a prognostic marker of hospital mortality in ARDS patients^51^. Another study also showed that the level of serum uric acid could be used to predict the severity and prognosis of septicemia^52^. Study had found that hyperuricemia can be used as a sign of poor prognosis and mortality in patients with sepsis. As a molecule, uric acid suggested the damage of energy metabolism and cellular protein catabolism, and may be used as a measure of sepsis status and progression^53^. In our study, it was found that hyperuricemia is associated with increased risk 90-day all-cause mortality of ICU patients with sepsis. Hyperuricemia may be an important prognostic marker for increased 90-day mortality. At the same time, we should pay attention to the occurrence of deaths of hyperuricemia patients with high risk factors in the subgroup, and take measures to prevent the deterioration of the condition.

In a study of 59,219 patients, it was found that serum uric acid may be an independent risk factor for AKI in all hospitalized patients^54^. It has been reported that increased uric acid levels in sepsis patients are associated with an increased risk of AKI^19^. Elevated serum uric acid levels in sepsis patients have also been shown to promote endothelial progenitor cell mobilization, leading to AKI^55^. In a meta-analysis, elevated serum uric acid levels were associated with an increased risk of AKI^56^. Study had found that the value of serum uric acid in predicting the occurrence of AKI after cardiovascular surgery was similar to conventional biomarkers and new biomarkers^57^. Interestingly, after matching, the incidence of AKI was higher in the hyperuricemia group than in the normal uric acid level group (19.1% vs. 14.7%, *p* = 0.046), and Hyperuricemia was related to the occurrence of AKI (OR 1.773, 95% CI 1.107–2.841, *p* = 0.017). In the subgroup analysis in this study, hyperuricemia was associated with an increased risk of AKI in patients aged > 65 years, males, patients with hypertension, patients with bilirubin > 0.9, patients with PT < 14.4, so we should pay attention to the impact of the risk factors in the above subgroups on the occurrence of AKI, so that we can take timely measures to prevent. Acute kidney injury is a common complication in sepsis, and its mortality rate is as high as 70%^58^. Study had also found that lowering uric acid may have a potential protective effect on kidney structural damage^59^. Therefore, in the early stage, drug intervention in serum uric acid levels may protect kidney function, thereby reducing the mortality of patients with sepsis.

There are several limitations of this study. First, this was a single-center retrospective study with possible selection bias, although we minimized differences in baseline clinical data through propensity score matching. Second, some unmeasured confounders, such as metabolic diseases, could have influenced the results. Third, as the data were obtained from a retrospective database, no prehospital interventions affecting the serum uric acid level were recorded. Finally, we cannot determine the underlying mechanism between high uric acid and prognosis. Therefore, we need to design a large multicenter prospective study to further confirm the above results and further study regarding the mechanism.

## Conclusion

In this large retrospective study, we confirmed that high serum uric acid levels were significantly associated with increased risk 90-day all-cause mortality and the incidence of AKI in sepsis patients in the ICU. Serum uric acid levels may be a useful biomarker for 90-day mortality and the incidence of AKI. However, our findings need to be further confirmed by large multicenter prospective studies, and additional studies need to be performed to identify the specific mechanism by which hyperuricemia affects the long-term prognosis of patients with sepsis.

## Supplementary Information

Supplementary Table S1.

## Data Availability

Original data used in this study is from the MIMIC-III database: MIMIC III (https://physionet.org/content/mimiciii/1.4/, version 1.4). The author (Z.Z.) obtained access to this database (certification number: 37242663) and was responsible for extracting the data. If needed, related data can be provided by contacting F.L. and Z.Z.
