# Supplementary material for: Prognostic value of hyperuricemia for patients with sepsis in the intensive care unit
Source: Sci Rep. 2022 Jan 20;12:1070. doi: 10.1038/s41598-022-04862-3 (PMC8776761; doi:10.1038/s41598-022-04862-3)
Supplement: Supplementary file 1 — Supplementary Table S1. [file 41598_2022_4862_MOESM1_ESM.docx]

Supplementary Table1. Univariate or multivariate analyses were used to assess the risk about association of hyperuricemia with primary and secondary outcomes.

| Clinical Outcomes | Before matching | | | | After matching | | | |
| --- | --- | --- | --- | --- | --- | --- | --- | --- |
|  | univariate | | multivariate | | univariate | | multivariate | |
|  | HR/OR(95%CI) | P value | HR/OR(95%CI) | P value | HR/OR(95%CI) | P value | HR/OR(95%CI) | P value |
| Length of stay | 0.991(0.980-1.002) | 0.124 | 0.983(0.969-0.998) | 0.022 | 1.004(0.986-1.021) | 0.689 | 1.003(0.983-1.024) | 0.740 |
| Hospital mortality | 1.736(1.349-2.236) | <0.001 | 1.605(1.197-2.151) | 0.002 | 1.409(1.002-1.979) | 0.048 | 1.554(1.091-2.214) | 0.015 |
| 30-day mortality | 1.634(1.305-2.046) | <0.001 | 1.628(1.256-2.109) | <0.001 | 1.435(1.055-1.950) | 0.021 | 1.712(1.239-2.364) | 0.001 |
| 90-day mortality | 1.537(1.249-1.892) | <0.001 | 1.475(1.162-1.872) | <.001 | 1.365(1.027-1.815) | 0.032 | 1.648(1.215-2.234) | 0.006 |
| AKI | 1.011(0.763-1.340) | 0.939 | 1.959(1.367-2.807) | <0.001 | 1.475(1.010-2.154) | 0.044 | 1.773(1.107-2.841) | 0.017 |
